# Profiles and predictors of healthcare utilization: using a cluster-analytic approach to identify typical users across conventional, allied and complementary medicine, and self-care

**DOI:** 10.1186/s12913-021-07426-9

**Published:** 2022-01-05

**Authors:** Daniela Rodrigues Recchia, Holger Cramer, Jon Wardle, David J. Lee, Thomas Ostermann, Romy Lauche

**Affiliations:** 1 Chair of Research Methods and Statistics in Psychology, Department of Psychology and Psychotherapy, Witten/Herdecke University, Alfred-Herrhausen-Strasse 50, 58448 Witten, Germany; 2 Department of Internal and Integrative Medicine, Evang. Kliniken Essen-Mitte, Faculty of Medicine, University of Duisburg-Essen, Essen, Germany; 3 National Centre for Naturopathic Medicine, Southern Cross University, Lismore, New South Wales Australia; 4 Chair of Department of Public Health Sciences, University of Miami, Miami, FL USA

**Keywords:** Complementary medicine, Cluster analysis, Healthcare utilization pattern

## Abstract

**Introduction:**

The identification of typologies of health care users and their specific characteristics can be performed using cluster analysis. This statistical approach aggregates similar users based on their common health-related behavior. This study aims to examine health care utilization patterns using cluster analysis; and the associations of health care user types with sociodemographic, health-related and health-system related factors.

**Methods:**

Cross-sectional data from the 2012 National Health Interview Survey were used. Health care utilization was measured by consultations with a variety of medical, allied and complementary health practitioners or the use of several interventions (exercise, diet, supplementation etc.) within the past 12 months (used vs. not used). A model-based clustering approach based on finite normal mixture modelling, and several indices of cluster fit were determined. Health care utilization within the cluster was analyzed descriptively, and independent predictors of belonging to the respective clusters were analyzed using logistic regression models including sociodemographic, health- and health insurance-related factors.

**Results:**

Nine distinct health care user types were identified, ranging from nearly non-use of health care modalities to over-utilization of medical, allied and complementary health care. Several sociodemographic and health-related characteristics were predictive of belonging to the respective health care user types, including age, gender, health status, education, income, ethnicity, and health care coverage.

**Conclusions:**

Cluster analysis can be used to identify typical health care utilization patterns based on empirical data; and those typologies are related to a variety of sociodemographic and health-related characteristics. These findings on individual differences regarding health care access and utilization can inform future health care research and policy regarding how to improve accessibility of different medical approaches.

## Introduction

The use of health services is a very individual process, yet it is shaped by institutional, cultural and social circumstances. Plenty of research has investigated determinants of health care use. Those include contextual characteristics and their influence on health care access [1], patient segmentation [2], digital interventions in relation to health behaviors [3] and the relation of physical activity and healthcare utilization [4] to predict health care utilization, and improve health care provision while at the same time targeting costs.

Research however often does not include the integrative approach of complementary medicine usage and user’s typology. So far, a comprehensive understanding of the patterns of health care use, and its individual determinants is missing. Examining health care utilization patterns may assist in understanding over- and underutilization, and related factors, and has the potential to shape health care provision, research and policy.

Analysis of health care utilization patterns has been advanced by new statistical methods and increasing computing power to employ more accurate and adequate (big) data analysis methods such as model-based cluster analysis and classification. Such methods have been used before to analyze homeless shelter utilization patterns [5], dietary [6] or other health related behavior patterns [7].

Cluster analysis is a statistical technique used to recognize natural patterns of subjects, i.e. to group them in such way that subjects in one group or cluster are more alike than subjects in different groups or clusters with regards to defined patterns, such as health care utilisation. Similarity of subjects can be defined by various methods and indices including the Euclidean distance between observations or the density estimation of subject distribution. Using this approach, one can identify empirical health care utilization patterns, and define typologies of consumers and their specific characteristics based on the pattern.

To our knowledge no study has determined general health care utilization patterns, this is, determined patterns of conventional, allied medicine, complementary medicine, and self-care in a nationally representative sample. While there is a study on pattern of complementary medicine use in the US-based National Health Interview Survey (NHIS) (2012), it was limited to children with mental health issues [8].

This study aims to examine NHIS health care utilization patterns in adults using a cluster analytic approach; and the associations of cluster patterns with sociodemographic, health-related and health-system related factors.

## Methods

The nationally representative NHIS monitors the health status and health care access and utilization of the non-institutionalized US population on a yearly basis including the use of complementary and alternative medicine (CAM) therapies every 5 years. For this analysis data from the Family Core, the Sample Adult Core, and the Adult Complementary and Alternative Medicine questionnaires from 2012 were merged. The more recent NHIS 2017 no longer assessed the totality of CAM modalities, but was limited to a few selected approaches. Thereby, data on the use of common treatment modalities such as acupuncture, osteopathy, supplements or herbal medicine were missing. We therefore chose to investigate the more comprehensive 2012 dataset.

The Family Core and the Sample Adult Core questionnaires collected data on socio-demographic characteristics including age, gender, ethnicity, region, marital status, education, and annual household income; self-perceived general health status, diagnosed conditions and diseases; and health care coverage, access and utilization. The Adult Complementary and Alternative Medicine questionnaire collected data on the use of complementary and alternative medicine.

Health care use in the past 12 months was queried with several question designs, such as whether a practitioner was consulted, or an intervention was used *(“During the past 12 months, did you see a practitioner/ use/ practice …?”*). Visits to the dentist and emergency room were queried differently, see Table 1. All items were coded as or recoded into binary variables (used vs. not used in the past 12 months) for the cluster analysis. A number of complementary and alternative therapies were combined to respond to the lack of observations. For example, all queried herbal medicines were combined into one variable (used herbal medicine vs. did not use herbal medicine); the same was done for non-vitamin supplements, vitamins and minerals, native healers, osteopathy and craniosacral therapy, Tai Chi and qigong, all forms of meditation, exercises and medical diets (see Table 1).

**Table 1:** Overview of questions related to health care utilization

ITEM	RELATED SURVEY QUESTION
Conventional health care provider	**DURING THE PAST 12 MONTHS, have you seen or talked to any of the following health care providers about your own health …**
GP	a general doctor who treats a variety of illnesses (a doctor in general practice, family medicine, or internal medicine)
Specialist	a medical doctor who specializes in a particular medical disease or problem (other than obstetrician/gynecologist, psychiatrist or ophthalmologist)
Eye specialist	an optometrist, ophthalmologist, or eye doctor (someone who prescribes eyeglasses
Mental health provider	a mental health professional such as a psychiatrist, psychologist, psychiatric nurse, or clinical social worker
Dentist	**About how long has it been since you last saw a dentist?** Include all types of dentists, such as orthodontists, oral surgeons, and all other dental specialists, as well as dental hygienists.
Emergency room	**DURING THE PAST 12 MONTHS, HOW MANY TIMES** have you gone to a HOSPITAL EMERGENCY ROOM about your own health
Allied health care provider	
Physical therapy	Physical therapist, speech therapist, respiratory therapist, audiologist, or occupational therapist
Nurse practitioners	Nurse practitioner, physician assistant, or midwife
CAM provider	**DURING THE PAST 12 MONTHS, did you see …**
Chiropractic	Practitioner for chiropractic
Massage	Practitioner for massage
Osteopathy, Craniosacral therapy*	Practitioner for osteopathic manipulation, for craniosacral therapy
Acupuncture	Practitioner for acupuncture
Naturopathy	Naturopath/a herbalist
Homeopathy	Homeopath
Ayurveda	Practitioner for Ayurveda
Native healing*	Native American Healer, Medicine Man, Shaman, Curandero, Machi, Parchero, Yerbero, Hierbista, Sobador, Huesero,
Energy healing	Provider or practitioner for energy healing therapy
Hypnosis	Practitioner for hypnosis
Exercise	**DURING THE PAST 12 MONTHS, did you practice …**
Yoga	Yoga
Tai Chi, Qigong*	Tai Chi/Qi Gong
Exercise*	Pilates, Trager Psychophysical Integration, Feldenkrais, Alexander Technique
Relaxation	**DURING THE PAST 12 MONTHS, did you use …**
Meditation*	Mantra Meditation, including Transcendental Meditation®, Relaxation Response, and Clinically Standardized Meditation; Mindfulness meditation, including Vipassana, Zen Buddhist meditation, Mindfulness-based Stress Reduction, and Mindfulness-based Cognitive Therapy; Spiritual meditation including Centering Prayer and Contemplative Meditation
Relaxation*	Guided imagery, progressive relaxation
Diet	**DURING THE PAST 12 MONTHS, did you use … for two weeks or more for health reasons**
Vegetarian diet*	Vegetarian diet, including Vegan diet
Medical diet*	Atkins diet, Ornish diet, Pritikin diet, Macrobiotic diet
OTC medication, supplements	**DURING THE PAST 12 MONTHS, did you take …**
Herbal medicine*	Combination herb pill, Acai pills or gel caps, Cranberry pulls or capsules, Echinacea, Garlic supplements, *Ginkgo Biloba*, Green rea pulls or EGCG pills, Milt thistle (sylimarin), Saw palmetto, Valerian, other herbs
Supplements*	Chondroitin, co-enzyme Q10, digestive enzymes, fish oil, glucosamine, melatonin, SAM-e, probiotics, other non-vitamin supplements
Vitamins minerals*	Multi-vitamins or multi-minerals, vitamins A,B,C,D,E,H, or K, calcium, magnesium, iron, chromium, zinc, selenium, or potassium

* indicates a merged variable

A total of 42,366 households were eligible and 34,525 adults provided data (79.7% response rate) [9]. The final analysis was conducted on 32,017 (75.6%) adults providing complete health care utilization data for all modalities. Population-based estimates were calculated using weights calibrated to the 2010 census-based population estimates for age, gender, and ethnicity of the US civilian non-institutionalized population. By using the population weights the full dataset of 34,525 adults represents a total of 234,9 million US adults.

### Statistical analysis

Distribution of frequencies of health care use and sociodemographic data within each cluster are presented as relative percentages (%) in Tables 2 and 3 respectively. To identify possible user typologies and their utilization pattern, the cluster analysis approach was used. This method is able to handle complex data structures and designs in big data scenarios.

**Table 2:** Prevalence of health care use within each cluster; presented in % used within cluster categories. Only those with a probability of ≥95% of belonging to each cluster have been selected. Numbers are presented in millions

	Average	Cluster 1	Cluster 2	Cluster 3	Cluster 4	Cluster 5	Cluster 6	Cluster 7	Cluster 8	Cluster 9
	*n* = 205.2	*n* = 31.3 15.3%	*n* = 35.1 17.1%	*n* = 15.4 7.5%	*n* = 26.7 13.0%	*n* = 20.2 9.8%	*n* = 25.4 12.4%	*n* = 15.0 7.3%	*n* = 18.1 8.8%	*n* = 18.1 8.8%
**Conventional health care provider**										
GP	66.9	95.3	24.1	0.0	100.0	0.0	100.0	0.0	0.0	100.0
Specialist	26.1	99.9	3.4	6.4	0.0	3.9	18.0	7.6	6.8	22.8
Eye specialist	37.8	77.5	0.0	18.3	100.0	10.0	38.4	18.9	27.3	27.2
Mental health provider	7.5	15.9	7.7	3.6	7.4	2.6	5.9	4.1	4.1	8.2
Dentist	61.3	73.5	63.8	0.0	75.1	0.0	100.0	100.0	100.0	0.0
Emergency room	19.4	36.2	17.9	12.3	14.5	11.6	16.9	10.1	9.0	29.1
**Allied health care provider**										
Physical therapy	8.6	30.6	5.5	2.1	6.9	1.3	4.5	2.0	2.4	6.4
Nurse practitioners	18.9	48.2	18.6	8.4	20.2	4.2	12.5	6.2	11.6	16.1
**CAM provider**										
Chiropractic	8.1	15.1	8.6	4.9	10.6	2.6	5.1	4.4	9.1	4.7
Massage	6.4	12.6	7.7	4.0	8.3	1.2	3.2	2.4	8.5	2.0
Osteopathy, Craniosacral	0.5	1.3	0.5	0.1	0.4	0.2	0.2	0.1	0.7	3.3
Acupuncture	1.5	3.3	1.6	0.7	1.9	0.4	0.6	0.9	1.7	0.3
Naturopathy	1.1	3.0	1.0	1.1	1.1	0.0	0.1	0.1	1.7	0.2
Homeopathy	0.4	0.6	0.6	0.5	0.4	0.0	0.0	0.1	0.4	0.1
Ayurveda	0.1	0.2	0.1	0.1	0.1	0.0	0.1	0.1	0.3	0.0
Native healing	0.4	0.4	0.4	1.1	0.4	0.4	0.2	0.2	0.4	0.1
Energy healing	0.4	1.0	0.4	0.4	0.5	0.0	0.1	0.2	0.8	0.1
Hypnosis	0.1	0.3	0.1	0.1	0.4	0.0	0.0	0.0	0.2	0.0
**Exercise**										
Yoga	8.2	11.4	10.0	8.8	11.5	2.2	4.3	4.3	14.0	2.5
Tai Chi, Qigong	1.2	2.3	1.1	1.6	2.0	0.3	0.4	0.2	1.4	0.4
Exercise	2.2	3.8	2.4	1.7	4.1	0.3	1.0	1.0	3.2	0.3
**Relaxation**										
Meditation	3.7	8.2	4.3	4.0	3.9	1.1	1.2	1.2	4.3	1.2
Relaxation	2.4	6.4	2.5	2.6	2.3	0.8	0.5	0.3	2.6	0.7
**Diet**										
Vegetarian diet	1.7	2.6	1.7	2.0	2.5	0.9	1.0	1.2	1.9	0.7
Medical diet	1.1	2.3	1.3	1.0	1.5	0.1	0.5	0.2	1.4	0.3
**OTC medication, supplements**										
Herbal medicine	7.6	13.6	9.5	12.0	10.2	1.1	1.4	1.2	11.9	28.9
Supplements	15.0	28.9	18.6	18.8	22.2	1.6	2.7	2.7	20.2	2.9
Vitamins minerals	61.7	94.7	100.0	100.0	100.0	0.0	0.0	0.0	100.0	0.0

**Table 3:** Associations between clusters and sociodemographic, health- and health-insurance related factors, in % within clusters

		Average	Cluster 1	Cluster 2	Cluster 3	Cluster 4	Cluster 5	Cluster 6	Cluster 7	Cluster 8	Cluster 9
Sociodemographics		*n* = 205.2	*n* = 31.3 15.3%	*n* = 35.1 17.1%	*n* = 15.4 7.5%	*n* = 26.7 13.0%	*n* = 20.2 9.8%	*n* = 25.4 12.4%	*n* = 15.0 7.3%	*n* = 18.1 8.8%	*n* = 18.1 8.8%
**Age, in years**	18–29	21.6	7.9	20.3	30.0	14.6	35.0	24.0	35.9	29.8	18.0
	30–39	16.7	9.6	18.8	22.6	11.7	21.8	17.5	19.7	22.0	16.3
	40–49	17.9	13.4	19.9	19.6	14.8	18.9	19.9	19.1	18.6	18.4
	50–64	25.8	35.1	26.3	19.8	29.7	18.6	24.4	17.9	22.2	25.1
	> 65	18.0	34.0	14.7	8.0	29.3	5.8	14.1	7.4	7.4	22.1
**Gender**	Male	49.3	42.5	43.3	56.1	38.8	64.5	54.4	58.8	47.5	54.4
	Female	50.7	57.5	56.7	43.9	61.2	35.5	45.6	41.2	52.5	45.6
**Ethnicity**	Non-Hispanic White	66.9	81.1	68.4	57.3	73.6	47.1	63.9	60.2	69.0	61.3
	Hispanic	15.3	7.3	13..8	23.2	9.9	30.2	15.5	18.9	14.2	17.9
	Black	11.8	8.1	11.7	11.9	10.3	15.3	14.1	13.4	9.8	15.5
	Asian	5.3	3.1	5.3	6.5	5.6	6.4	5.6	6.6	6.5	4.2
	Other	0.8	0.4	0.7	1.1	0.6	1.0	0.9	0.9	0.5	1.1
**Region**	West	22.5	21.1	25.0	28.3	22.4	23.2	18.5	21.4	27.5	17.6
	Northeast	18.2	20.3	17.7	11.6	20.0	12.8	21.7	22.5	16.4	17.4
	Midwest	22.5	24.6	22.0	19.1	24.1	20.0	23.6	21.5	23.0	21.5
	South	36.7	34.1	35.3	41.0	33.5	44.1	36.3	34.6	33.2	43.4
**Education**	Less than high school	14.0	8.0	12.5	17.0	8.7	28.2	12.3	14.1	8.6	28.6
	High school	47.2	43.0	46.8	52.8	43.9	53.2	50.3	48.3	42.4	50.3
	College or more	38.8	48.9	40.7	30.2	47.4	18.6	37.5	37.6	49.1	21.1
**Employment**	not employed	33.8	45.5	30.8	26.3	37.4	30.1	32.4	24.3	20.5	45.4
	employed	66.2	54.5	69.2	73.7	62.6	69.9	67.6	75.7	79.5	54.6
**Income**	< 20.000	30.7	24.2	28.6	36.3	26.1	49.3	25.0	31.4	28.4	39.6
	20.000–34.999	21.5	18.4	21.1	28.6	17.8	25.5	21.1	19.3	21.1	27.8
	35.000–64.999	28.0	30.3	29.0	24.5	29.8	19.3	30.0	29.7	30.1	23.0
	> 65.000	19.8	27.0	21.3	10.6	26.3	5.9	23.9	19.6	20.3	9.6
**Marital status**	in relationship	39.6	33.3	37.7	45.0	37.6	48.6	39.3	44.9	37.9	43.1
	not in relationship	60.4	66.7	62.3	55.0	62.4	51.4	60.7	55.1	62.1	56.9
**BMI**	18.5 to 25	33.4	29.7	33.6	37.9	33.6	34.2	32.8	37.0	40.7	25.5
	up to 18.5	1.7	1.3	1.8	1.4	1.5	1.7	1.6	1.7	2.1	2.5
	25–30	33.9	34.4	33.7	33.5	35.2	32.8	32.6	34.8	34.6	34.1
	30 and more	31.0	34.7	30.9	27.2	29.8	31.3	33.0	26.5	22.6	37.9
**Health status**	very good/excellent	60.1	45.4	62.9	62.4	65.1	59.5	65.5	72.2	77.5	41.0
	good	26.8	30.9	26.5	28.0	26.2	28.7	25.0	21.4	18.8	32.2
	fair to poor	13.0	23.7	10.5	9.6	8.7	11.8	9.5	6.4	3.7	26.8
**Chronic Conditions**	no chronic condition	56.1	27.8	57.9	74.1	47.1	78.9	58.4	78.0	77.5	44.3
	one chronic condition	23.4	27.5	25.7	17.7	27.3	14.8	25.6	17.2	16.7	25.2
	two chronic conditions	11.6	21.0	10.3	6.1	15.6	4.1	10.5	3.6	4.5	15.5
	≥ three chronic conditions	8.8	23.7	6.0	2.1	10.0	2.2	5.5	1.3	1.3	15.0
**Smoking**	Non-smoking	81.9	88.3	82.8	76.1	87.9	68.3	84.6	81.8	85.7	70.5
	Smoking	18.1	11.7	17.2	23.9	12.1	31.7	15.4	18.2	14.3	29.5
**Alcohol**	Abstainers	35.9	35.8	32.8	33.4	33.1	42.0	38.4	36.5	28.9	47.8
	Light	43.7	45.2	46.5	44.6	46.5	37.3	42.4	42.9	47.5	36.4
	Moderate to heavy	20.3	18.9	20.8	22.0	20.4	20.8	19.3	20.6	23.6	15.8
**Exercise**	Sedentary	80.4	83.0	78.9	75.9	79.1	84.2	81.6	77.2	73.9	87.6
	Moderate level	14.5	13.4	15.8	17.4	15.5	10.4	13.2	16.3	20.4	8.4
	High level	5.1	3.7	5.3	6.7	5.3	5.4	5.1	6.5	5.7	4.0
**Health insurance**	no insurance	17.3	3.4	13.1	43.1	5.7	53.4	8.5	19.6	17.6	21.4
	public	19.3	26.2	18.3	12.9	19.7	14.0	19.2	14.0	9.5	33.9
	private	63.4	70.3	68.5	44.0	74.5	32.7	72.4	66.4	72.9	44.7
**Could not afford**	Prescription medication	8.2	9.6	8.5	13.2	5.2	10.9	4.6	4.0	4.5	14.3
	Mental Care	2.4	3.2	2.3	4.3	1.1	2.9	1.2	1.9	1.7	3.4
	Dental Care	13.1	12.6	14.7	23.3	8.5	20.1	6.6	7.9	8.7	19.2
	Eyeglass	7.8	8.2	9.1	13.5	4.7	11.0	4.1	3.8	5.0	12.1
	Specialist	4.9	6.1	5.0	9.2	2.4	6.5	2.2	2.6	3.1	7.3

The cluster analysis was performed using binary variables only, i.e. all modalities were coded as “*used in the past 12 months”* vs. *“not used in the past 12 months”*, independent of the frequency of use. For the ease of interpretation, cluster analyses based on probabilities are preferred over other models [10, 11]; thus, a model-based clustering using finite normal mixture modelling was chosen. This method provides functions for parameter estimation via the Expectation Maximization (EM) algorithm for normal mixture models with the possibility of accounting for different covariance structures and the integration of the Bayesian Information Criterion (BIC) for model selection.

The cluster analysis was performed using the package mclust [12] for the statistical software R [13], this package also handles sampling weights if needed. The fitted model was specified as *“EII” – spherical, equal volume multivariate mixture*, which specifies a spherical distribution with both volume and shape equal enabling the parameters to be better estimated. These parameters consider the within-group covariance matrix.

To determine the statistical fit of the respective cluster solutions the following indices were independently determined: Bayesian Information Criterion (BIC) [14, 15], the Dunn Index [16], Silhouette [17], the Davies-Bouldin index [18], and the C-index [19]. The following order by which the results of the cluster analysis were valuated was used: Silhouette Width, C-index and Dunn-index, BIC and Davies-Bouldin index, these indices are presented at Table 5.

The cluster solutions also contained information on the probabilities with which the person fit into the cluster. They are based on a classification matrix generated by the cluster analysis. This matrix contains the probability that the observation belongs to each cluster. From this classification matrix the uncertainty values were defined and only those subjects who had a 95% probability of belonging to the respective cluster (*n* = 30,251; 87.6%) were considered for further analysis.

Sociodemographic, health related characteristics and health care access were compared between the respective clusters. The variable classes were based on a similar study from NHIS [20]. The following sociodemographic predictors were considered: age (categories: 18–29; 30–39; 40–49; 50–64, 65 or older), gender (categories: female; male), ethnicity (categories: non-Hispanic White; Hispanic; African American; Asian; Other), US region (categories: West; Northeast; Midwest; South), marital status (categories: not in relationship; in relationship), education (categories: less than college; some college or more), employment (categories: employed, not employed) and annual household income (categories: less than $20,000; $20,000 to $34,999; $35,000–$64,999; $65,000 or more). Additionally, health related factors such as general health status (categories: excellent, very good, good, fair or poor), medical conditions/diseases (no chronic condition, one chronic condition, two chronic conditions, three or more chronic conditions), BMI (categories: < 18.5; 18.5–25; 25.5–30; 30.5 or more), health behaviors such as smoking (categories: non-smoker, smoker), alcohol consumption (categories: alcohol abstainer; light drinker; regular or heavy drinker), and exercise behavior (categories: low level exerciser, moderate level exerciser, high level exerciser); health insurance coverage (categories: no insurance, public health insurance, private health insurance) and the affordability of prescription medication, mental care, dental care, eyeglasses and specialists (categories: could afford, could not afford) were also used as potential predictors.

Backward stepwise regression analyses employing a likelihood-ratio-statistic were conducted for each cluster to determine predictors for belonging in that cluster, and adjusted odds ratios with 95% confidence intervals were calculated. All potential predictors were included in the logistic regression analyses, and a sample size adjusted weight was used considering for design effects. Statistical significance was set at *p* ≤ 0.05.

The regression models and distributions analyses were performed with Statistical Package for Social Sciences software (IBM SPSS Statistics for Windows, release 22.0. Armonk, NY: IBM Corp.).

## Results

A total of 32,017 of 34,525 subjects (92.7%) provided full data on health care use and therefore were selected for the cluster analysis, 30,251 (87.6%) were considered for the estimation of sample representativeness. Based on the population weight the sample of 30,251 sample represents a total of 205.2 million US adults.

Based on the individual health care utilization patterns, the optimal cluster solution as per Silhouette and the C-index was identified as having 9 clusters (Table 5), with cluster size ranging from 2067 to 5117 observations. The model fit indices were 0.21 for Silhouette, and 0.24 for C-index, indicating sufficient statistical fit.

**Fig. 1:**
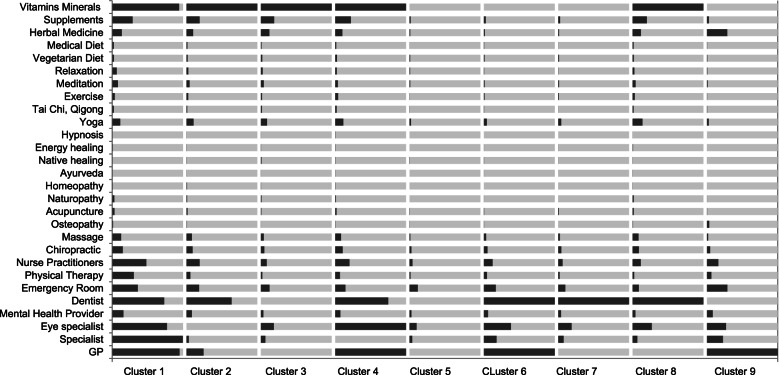
Visualization of health care utilization patterns within each cluster. Within each cluster dark color indicates use in %, bright color non-use. Sizes of clusters are not indicative of actual size

The prevalence of health care utilization within each cluster are shown in Table 2. Sociodemographic, health- and health-insurance related associations for belonging in either cluster can be found in Tables 3 and 4. A graphic visualization from health care utilization patters within each cluster is presented in Fig. 1. The following clusters of health care user types have been identified, and analyzed with regards to their characteristics. The respective frequencies are displayed at Table 3.

**Table 4:** Predictors as per logistic regression, presented as Odds Ratio (95% Confidence Interval). All Odds Ratios significant at *p* < 0.05

		Cluster 1	Cluster 2	Cluster 3	Cluster 4	Cluster 5	Cluster 6	Cluster 7	Cluster 8	Cluster 9
Sociodemographics		*n* = 31.3 15.3%	*n* = 35.1 17.1%	*n* = 15.4 7.5%	*n* = 26.7 13.0%	*n* = 20.2 9.8%	*n* = 25.4 12.4%	*n* = 15.0 7.3%	*n* = 18.1 8.8%	*n* = 18.1 8.8%
**Age, in years**	18–29	Reference	Reference		Reference	Reference	Reference	Reference	Reference	Reference
	30–39	1.09 0.90; 1.31	1.23 1.10; 1.39		0.89 0.75; 1.05	1.10 0.94; 1.29	0.98 0.85; 1.13	0.74 0.63; 0.86	0.96 0.83; 1.11	1.21 1.00; 1.45
	40–49	1.15 0.96; 1.38	1.19 1.05;1.34		1.13 0.96; 1.33	1.00 0.84; 1.18	1.02 0.88; 1.17	0.79 0.68; 0.92	0.84 0.73; 0.98	1.23 1.02; 1.49
	50–64	1.68 1.41; 1.99	1.12 0.99; 1.27		1.40 1.20; 1.64	0.98 0.82; 1.17	0.86 0.74; 0.99	0.57 0.48; 0.68	0.88 0.76; 1.03	0.98 0.81; 1.20
	> 65	2.45 1.97; 3.06	0.88 0.70; 1.10		2.01 1.61; 2.50	0.49 0.31; 0.77	0.49 0.37; 0.65	0.37 0.25; 0.57	0.68 0.50; 0.93	0.73 0.52; 1.02
**Gender**	Male	Reference	Reference	Reference	Reference	Reference	Reference	Reference	Reference	Reference
	Female	1.49 1.35; 1.65	1.30 1.20; 1.41	0.82 0.72; 0.92	1.72 1.55; 1.91	0.43 0.38; 0.49	0.77 0.70; 0.85	0.67 0.59; 0.75	1.21 1.09; 1.34	0.62 0.55; 0.71
**Ethnicity**	Non-Hispanic White	Reference		Reference		Reference	Reference	Reference		
	Hispanic	0.59 0.49; 0.71		0.88 0.75; 1.03		1.35 1.16; 1.57	1.32 1.14; 1.53	1.25 1.07; 1.46		
	Black	0.62 0.52; 0.74		0.90 0.74; 1.09		1.41 1.18; 1.68	1.22 1.05; 1.42	1.13 0.93; 1.36		
	Asian	0.44 0.34; 0.58		1.31 1.03; 1.67		1.61 1.25; 2.06	1.19 0.96; 1.48	1.38 1.09; 1.75		
	Other	0.38 0.16; 0.89		1.37 0.79; 2.40		1.48 0.85; 2.59	1.24 0.68; 2.26	1.14 0.56; 2.35		
**Region**	West		Reference	Reference			Reference	Reference	Reference	Reference
	Northeast		0.84 0.74; 0.96	0.50 0.41; 0.62			1.62 1.39; 1.89	1.54 1.29; 1.85	0.70 0.59; 0.82	1.33 1.09; 1.63
	Midwest		0.81 0.72; 0.91	0.68 0.57; 0.80			1.33 1.15; 1.55	1.18 0.99; 1.41	0.91 0.79; 1.04	1.27 1.05; 1.53
	South		0.87 0.79; 0.97	0.87 0.75; 1.00			1.32 1.15; 1.52	1.18 1.00; 1.39	0.83 0.73; 0.95	1.41 1.19; 1.67
**Education**	Less than high school	Reference		Reference	Reference	Reference	Reference		Reference	Reference
	High school	2.18 1.67; 2.86		1.05 0.87; 1.26	1.81 1.42; 2.31	0.63 0.54; 0.73	1.32 1.09; 1.60		1.09 0.90; 1.33	0.72 0.61; 0.86
	College or more	3.31 2.52; 4.35		0.79 0.64; 0.97	2.07 1.61; 2.66	0.39 0.32; 0.47	1.08 0.89; 1.32		1.33 1.09; 1.62	0.55 0.45; 0.67
**Income**	< 20.000	Reference		Reference	Reference	Reference				Reference
	20.000–34.999	1.08 0.93; 1.26		1.37 1.18; 1.58	0.87 0.75; 1.02	0.78 0.68; 0.90				1.07 0.91; 1.25
	35.000–64.999	1.15 0.99; 1.33		1.14 0.96; 1.34	0.95 0.82; 1.10	0.68 0.57; 0.80				0.76 0.63; 0.91
	> 65.000	1.31 1.11; 1.54		0.84 0.71; 0.91	1.17 1.00; 1.38	0.39 0.30; 0.50				0.51 0.40; 0.65
**Marital status**	in relationship	Reference		Reference	Reference	Reference				
	not in relationship	1.22 1.10; 1.37		0.81 0.71; 0.91		0.86 0.77; 0.98				
**BMI**	18.5 to 25					Reference	Reference		Reference	Reference
	up to 18.5					0.77 0.47; 1.26			1.23 0.83; 1.83	1.57 0.99; 2.50
	25–30					1.05 0.92; 1.21			1.05 0.93; 1.18	1.22 1.04; 1.43
	30 and more					1.20 1.03; 1.38			0.86 0.75; 0.98	1.31 1.12; 1.54
**Health status**	very good/excellent	Reference		Reference	Reference		Reference	Reference	Reference	Reference
	good	1.33 1.18; 1.49		1.19 1.04; 1.36	0.93 0.83; 1.05		0.81 0.72; 0.91		0.71 0.62; 0.81	1.33 1.16; 1.53
	fair to poor	2.18 1.81; 2.63		0.99 0.77; 1.27	0.54 0.42; 0.71		0.77 0.61; 0.97		0.38 0.27; 0.55	1.75 1.42; 2.16
**Multiple chronic conditions**	no condition	Reference	Reference	Reference	Reference	Reference	Reference	Reference	Reference	Reference
	one condition	1.78 1.58; 2.00	1.10 1.00; 1.22	0.51 0.43; 0.60	1.23 1.09; 1.38	0.58 0.49; 0.68	1.20 1.07; 1.35	0.63 0.53; 0.74	0.59 0.51; 0.68	1.19 1.03; 1.38
	two conditions	2.60 2.22; 3.03	0.97 0.83;1.13	0.50 0.39; 0.66	1.28 1.08; 1.52	0.30 0.21; 0.42	0.93 0.76; 1.13	0.27 0.18; 0.40	0.39 0.29; 0.51	1.17 0.93; 1.47
	≥ three conditions	4.76 3.89; 5.82	0.58 0.44; 0.75	0.15 0.08; 0.29	1.07 0.82; 1.39	0.33 0.22; 0.51	0.69 0.50; 0.96	0.18 0.09; 0.36	0.18 0.10; 0.34	1.43 1.07; 1.90
**Smoking**	Non smoking	Reference			Reference	Reference			Reference	Reference
	Smoking	0.59 0.51; 0.69			0.81 0.70; 0.94	1.79 1.57; 2.04			0.83 0.72; 0.96	1.51 1.31; 1.74
**Alcohol consumption**	Abstainers	Reference	Reference		Reference	Reference	Reference			Reference
	Light	1.16 1.03; 1.31	1.20 1.09; 1.33		1.21 1.07; 1.36	0.76 0.67; 0.87	0.80 0.71; 0.89			0.82 0.71; 0.95
	Moderate to heavy	1.06 0.92; 1.23	1.20 1.07; 1.35		1.17 1.01; 1.35	0.87 0.74; 1.01	0.81 0.71; 0.93			0.78 0.65; 0.93
**Exercise**	Sedentary		Reference	Reference	Reference	Reference	Reference		Reference	Reference
	Moderate level		1.00 0.90; 1.12	1.28 1.11; 1.48	1.11 0.98; 1.26	0.65 0.55; 0.77	0.71 0.62; 0.82		1.30 1.15; 1.47	0.75 0.62; 0.90
	High level		1.25 1.06; 1.48	1.19 0.95; 1.49	1.27 1.04; 1.56	0.76 0.60; 0.95	0.80 0.65; 0.99		0.87 0.69; 1.09	0.77 0.58; 1.02
**Health insurance**	no insurance	Reference	Reference	Reference	Reference	Reference	Reference			Reference
	public	3.76 2.87; 4.92	1.45 1.21; 1.74	0.30 0.23; 0.39	2.24 1.74; 2.89	0.32 0.26; 0.40	3.33 2.63; 4.22			1.43 1.15; 1.79
	private	3.37 2.68; 4.24	1.54 1.36; 1.74	0.36 0.31; 0.41	2.45 2.00; 2.99	0.27 0.23; 0.31	3.15 2.61; 3.79			0.99 0.84; 1.17
**Could not afford**	Prescription medication		1.22 1.03; 1.44			0.73 0.60; 0.90		0.67 0.48; 0.94		1.45 1.20; 1.76
	Mental Care					0.65 0.44; 0.97				
	Dental Care		1.30 1.14; 1.49	1.64 1.42; 1.90	0.71 0.58; 0.87		0.56 0.45; 0.69	0.69 0.54; 0.88	0.68 0.57; 0.82	
	Eyeglass				1.34 1.06; 1.69		0.69 0.51; 0.92	0.70 0.50; 0.99		
	Specialist	1.86 1.48; 2.34

Cluster 1 – *“Overutilization of health care*”: White (81.1%), female (57.5%), higher educated (48.9%), and/or older adults (65 years or more; 34%) with higher income > $65,000 (27%), multiple chronic conditions (23.7%), and/or private health insurance (70.3%) more likely over-utilize every health care modality relative to the general population. This includes conventional, allied and complementary health care, and non-provider-based health care (i.e. self-care) (Table 2). Region, BMI and frequency of exercise were not relevant variables in the logistic model (Table 4).

Cluster 2 – *“High users of vitamins and mineral supplementation”*: Mid-aged (50–64 years old; 26.3%), female adults (56.7%) living in the Southern region of the country (35.3%) with one chronic condition (57.9%), and/or private health insurance (68.5%) more likely show average health care utilization in general. Relative to the general population they have lower consultation rates with GPs and specialists, and higher utilization of vitamins and mineral supplementation (Table 2). Ethnicity, Education, Income, Marital and Health Status, BMI and Smoking habits were not included in the final regression model (Table 4).

Cluster 3 – *“Underutilization of health care, GP, specialists, ER and dentists”*: Single (55%), male (56.1%) adults living in the South (41%) with high school but not college education (52.8%), small income (< $20,000; 36.3%), no chronic conditions (74.1%), and/or average health status (28%), but no health insurance (43.1%) more likely belonged to Cluster 3. Compared to Cluster 1 and 2, adults of Asian ethnicity also more likely belonged to this cluster (6.5%). Adults in this cluster underutilize conventional health care, including visits to GPs, specialists, ER and dentists relative to the general population. In contrast, health care interventions and behavior not associated with visits to a health professional are used on an average level (Table 2). This is the only cluster where age was not a relevant variable for the statistical model, together with BMI and Smoking and Drinking habits (Table 4).

Cluster 4 – *“Underutilization of specialists and emergency rooms”*: Female (61,2%), higher educated (47.4%), and/or older adults (> 65 years or more; 29.3%) with very high income >$65,000 (26.3%), one or two chronic conditions (42.9%), and/or private health insurance (74.5%) more likely show a high utilization of GPs, eye specialists, manual therapies and self-care. They are however characterized by an under-utilization of specialists visits and visits to the emergency room (Table 2). Ethnicity, Region and BMI were not present at the final logistic model (Table 4).

Cluster 5 – *“Underutilization of every health care modality”*: Young (35%), male (64.5%), and/or non-White individuals (52.9%) without high-school education (28.2%), very low income (49.3%), no health insurance (53.4%), and/or no chronic condition (78.9%) more likely show under-utilization of every health care modality (Table 2).

Cluster 6 – “*Underutilization of CAM and self-care”*: Young or mid-aged (41.5%), male (54.4%), and/or non-White individuals (36.1%), not living in the West (81.5%), having at least high-school education (50.3%), with good to very good health (65.5%) despite one chronic condition (58.4%) more likely show an over-utilization of GPs and dentists, average utilization of other conventional and allied health care, and an under-utilization of CAM and self-care (Table 2). Region, Health Status and Smoking habits were not relevant in the model (Table 4).

Cluster 7 – “*Underutilization of every health care but dental care”:* Very young (35.9%), male (58.8%), and/or non-White individuals (39.8%), not living in the West (78.6%), with no chronic condition (78%) more likely show under-utilization of every health care modality with the exception of dental care (Table 2). Education, Income, Marital and Health Status, BMI, Health Insurance and Smoking habits were not relevant (Table 4).

Cluster 8 – *“Underutilization of every health care modality”*: Very young (29.8%), female adults (52.5%), living in the West (27.5%), with higher education (49.1%), very good health (77.5%) and/or no chronic conditions (77.5%) more likely show under-utilization of every health care modality, and over-utilization of dental care and vitamin/mineral supplementation (Table 2). Ethnicity, Income, Health Insurance and Alcohol Consumptions were not included in the final model (Table 4).

Cluster 9 – *“Overutilization of GPs, ER and herbal medicines”*: Young to mid-aged (34.3%), male individuals (54.4%) not living in the West (82.4%), with no high-school or college education (71.4%), and/or with very low income (39.6%), poor health status (26.8%) with at least one chronic condition (25.2%), and/or public health insurance (33.9%) more likely show over-utilization of GPs, emergency rooms, and herbal medicines, and under-utilization of all other health care modalities (Table 2). For Cluster 9 only Ethnicity was not considered in the logistic model (Table 4).

## Discussion

### Summary of results

The cluster analysis identified several types of health care user based on their health care utilization patterns. However, no single optimal cluster solution could be consistently favored by all indices. The preferred cluster solution identified 9 types of health care users, who showed significant differences in the utilization patterns with substantial rates of over- and underutilization of certain health care modalities. The regression analysis further found sociodemographic, health and health insurance related factors predictive of being a member of a respective cluster. The presence or non-presence of multiple chronic conditions was the only variable identified in the logistic model to be significant as a predictor for all 9 clusters.

### Model fit and indices

The cluster analysis was performed with different numbers of clusters until the optimal final 9-cluster solution was identified. Because the dataset is relatively big, both regarding observations and variables, and the used method (mclust) iterates many parameters and run different estimations, the run time per adjusted model was considerably high (several days considering all models).

The final solution was achieved after evaluating for both statistical model properties – the cluster validation measures in Table 5, and for the theoretical interpretation of the similarity of characteristics. There was unfortunately not a single solution where all five statistical indices were optimal.

**Table 5:** Cluster solutions and their respective model fit according to different indices. Arrow indicates whether higher (↑) or lower scores (↓) are indicative of better model fit, * indicates best model fit for each of the indices

	Index				
Cluster solution	Silhouette ↑	C-index ↓	Dunn index ↑	BIC ↓	Davies-Bouldin index ↓
2 Clusters	0.14	0.37	0.20	885,194.80	2.58
3 Clusters	0.12	Inf	0.20	1,063,109.00*	3.75
4 Clusters	0.14	0.29	0.21	−80,894.13	1.95
5 Clusters	0.17	0.28	0.21	−41,601.08	1.81
6 Clusters	0.15	Inf	0.22*	− 8587.70	1.70
7 Clusters	0.18	0.24*	0.21	11,532.30	1.65*
8 Clusters	0.20	0.25	0.21	36,644.75	1.72
9 Clusters	0.21*	0.24*	0.21	54,450.64	1.75

In a review of clustering methods, mclust has shown a better overall cluster performance and ability regarding handling different data types [21]. This enhances a good level of certainty regarding the chosen method. The method incorporates the complex sampling design of the NHIS enabling a better model adequacy.

### Typology of health care users, and their impact

The cluster analysis identified 9 different types of health care users, who showed substantial differences in utilization of certain health care modalities. Members in the first cluster for example showed a substantial overutilization of practically every health care modality, while those in the fifth cluster were using almost no health care at all. Several sociodemographic and health-related characteristics were predictive of belonging into the respective clusters, including age and gender, education, income, ethnic origin, health care coverage, and health status. Several findings deserve attention. Below we summarized the findings into groups of differences and commonalities between the profiles of health care utilization (i.e. the clusters).

### Healthy aging

Members of the first cluster showed overutilization of health care, and that might be explained by the fact that they were more likely to be above 50 years of age and to have multiple chronic conditions. Chronic medical conditions are increasingly prevalent among older adults [22]. However, health status alone cannot fully explain the overutilization of health care modalities. Members in cluster 4, for example, also are more likely to be at the same age range, and to have chronic conditions. However, they are using far less practitioner-based health care modalities as compared to those in cluster 1. Clusters 1 and 4 probably reflect ‘more successful’ and ‘less successful’ pathways to aging. They also represent a substantial proportion of the US population, making this finding even more important given the aging of the US population (and in other industrialized nations). It has been known for some time that a small proportion of Americans account for the majority of healthcare expenditures and there are concerted efforts to better manage this utilization pattern [23]. Additionally, lifestyle factors that contributed to healthy aging such as non-smoking and social support [24], physical activity and diet quality [25] should be in focus of the coordination of public health care.

Clusters 2, 6 and 9 are mid-aged, and are likely to suffer from one or two (early stage) chronic conditions; individuals in cluster 2 strongly use self-care, those in 6 and 9 strongly utilize more conventional practitioner-based health care. We could not surely predict where these individuals would end up in higher age, and only taking action in time might prevent them from ending up in the over-user group.

### CAM

Cluster 6 shows an underutilization of CAM while an overutilization could not be detected in any cluster. This might indicate that we need to have a closer look at the efficacy and dissemination of CAM in the population. A program that would encourage the use of evidence-based CAM approaches such as the one applied for veterans [26] could be applied.

### Gender influence and self-care

We identified gender differences regarding health- and self-care behavior. Female individuals are more likely to use health-care in general (Cluster 1) or specifically vitamins & minerals supplementation (Cluster 2). On the other hand, males often underutilize health-care (Clusters 3 and 7) and self-care (Cluster 5). Research has shown consistently that men tend to neglect self-care [27] and engage less in health-related self-care behaviors [28]. From a public health perspective it is essential to raise awareness for the need of self-care in male populations.

Several factors might limit self-care besides gender: education/health literacy, self-efficacy, access or costs. Barriers need to be identified, and attempts to increase self-care utilization for improving overall health, preventing chronic conditions, and lower the costs associated with health care are needed.

### Healthcare coverage

A substantial proportion of participants (43.1% in Cluster 3 and 53.4% in Cluster 5) reported to have no health insurance. Clusters 4 (74.5%), 6 (72.4%) and 8 (72.9%) have the biggest proportion of private health insurance coverage. Other factors associated with significantly higher health-care use or problems when underserving those in need should be identifiable. Health status is reported as very good or excellent for a large proportion of participants in cluster 7 (72.2%) and 8 (77.5%), indicating a probable association with health insurance type.

### Preventive, curative or aesthetic intervention

Several health care interventions may not be related to a medical condition, but to preventative or aesthetic needs. Dentist visits for example are often for prevention, or for aesthetic purposes. Men were found to value dental care (Clusters 3 and 7) despite appearing ‘less caring’ about other health-care interventions. Dental care however is expensive, and not surprisingly, the highest frequency of participants who did not utilize dental care as frequently where those having no health insurance (Clusters 3 and 5).

### Implications for research

Cluster analyses have been used before in health research, for example in research of homeless shelter utilization patterns [5]. Several types of homeless people have been identified (e.g. transitionally homeless, episodically homeless, chronically homeless), and those types were associated with different usage patterns.

In this analysis, we used a model-based clustering approach based on finite normal mixture modelling, and the model was evaluated with several indices of cluster fit. This method has shown to be effective in differentiating and clustering individuals in 9 different groups with similar characteristics within and dissimilarities between clusters.

The findings of this cluster analysis may have important implications for health policy. They highlight distinct patterns of health care over- and underutilization associated with age, gender, socio-economical, ethnical and regional differences. By understanding health care utilization, interventional programs and prevention campaigns may be better tailored to specific groups of individuals with specific health care use patterns. Specifically, social inequalities and barriers to health care access can be addressed by tailoring health care to these groups.

The usage of CAM or dental care, where gender differences exist regarding self-care behavior, are areas that require attention. More awareness on the importance of health-care and program development to encourage and enable their utilization are crucial.

### Limitations

The findings identified through this analysis must be considered in light of the study limitations. The data were drawn from a cross-sectional survey; as such, the results can only suggest associations for a particular time point. A longitudinal survey would be necessary to document changes in health care utilization patterns over time.

Health care utilization was further queried using binary variables only, i.e. it was only assessed whether participants had used a certain health care intervention or not; and no information on the number of consultations, or the out of pocket expenditure was analyzed. Influence of binary variables on the cluster analyses, and distribution (low prevalence for several interventions) could not be measured regarding its frequency but only regarding its presence. The survey is further based on self-report data and as such there is at risk of recall bias or measurement error.

The decision in favor of the 9-cluster solution was not only based on statistical indices but on theoretical interpretation as well. There were different solutions according to the indices, indicating that there is not one solution, but several possibilities and the selection of cluster specification and its indices was user determined. Unfortunately an optimal single solution, where all 5 indices fit the best, could not be achieved.

Nevertheless, the US National Health Survey is an internationally recognized epidemiological study, and the findings from this study provide useful first insights into the patterns of health care utilization.

## Conclusions

In this analysis, we identified 9 types of health-care utilization patterns and their characteristics based on the similarity of their behavior. A model-based clustering approach based on finite normal mixture modelling and cluster fit indices were determined.

The clusters differentiate between health-care user types, ranging from nearly non-use of health care modalities to overutilization pattern of medical, allied and complementary health care. Several sociodemographic and health-related characteristics were predictive of belonging into a respective cluster, including age and gender, health status, education, income, ethnic origin, and health care coverage.

In conclusion, cluster analysis may be useful to identify typical health-care utilization patterns based on empirical data; and those typologies appear to be related to a variety of sociodemographic and health-related characteristics. Those findings can inform future health research and policy.

## Data Availability

The datasets analyzed are available at the open source from National Center for Health Statistics: https://www.cdc.gov/nchs/nhis/nhis_2012_data_release.htm.
